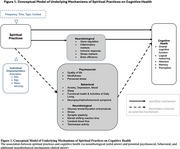# Potential Mechanisms Explaining Effects of Spiritual Practices on Cognitive Aging: A Systematic Review and Conceptual Model

**DOI:** 10.1002/alz70858_107459

**Published:** 2025-12-25

**Authors:** Katherine Carroll Britt, Hayoung Oh, Augustine Cassis Boateng, Sato Ashida, Roland J. Thorpe, Corey Nagel, Harold G. Koenig

**Affiliations:** ^1^ The University of Iowa College of Nursing, Iowa City, IA, USA; ^2^ Baylor University, Waco, TX, USA; ^3^ the University of Pennsylvania, Philadelphia, PA, USA; ^4^ University of Iowa College of Public Health, Iowa City, IA, USA; ^5^ Johns Hopkins Alzheimer's Disease Resource Center for Minority Aging Research, Bloomberg School of Public Health, Baltimore, MD, USA; ^6^ Johns Hopkins University, Baltimore, MD, USA; ^7^ University of Arkansas for Medical Sciences Colleges of Nursing & Public Health, Little Rock, AR, USA; ^8^ Duke University Medical Center, Durham, NC, USA; ^9^ King Abdulaziz University, Jeddah, Mecca Province, Saudi Arabia; ^10^ Shiraz University, Shiraz, Fars Province, Iran (Islamic Republic of)

## Abstract

**Background:**

Spiritual practices (i.e., practices that provide meaning, reflection, and personal connection with something beyond the physical realm) are a low‐cost, easily accessible therapeutic resource supporting cognitive health. Culturally relevant spiritual practices have the potential to optimize cognitive aging and reduce racial/ethnic disparities in cognitive health. Despite the increase in studies reporting cognitive health benefits of spiritual practices, the underlying mechanisms, which could enhance the impact and delivery of behavioral interventions, have not been thoroughly explored.

**Methods:**

We conducted a systematic literature review to summarize the evidence that may help to explain the underlying mechanisms by which spiritual practice associations affect cognitive health, thereby supporting a proposed conceptual model. We searched six databases (PubMed, PsychINFO, Embase, Sociological Abstracts, ATLA, CINAHL) and identified 34 original research studies published between 2000‐2023 that included spiritual practices, underlying mechanisms, and cognitive health (i.e., overall cognitive function and specific cognitive domains).

**Results:**

The included studies examined the spiritual practices of meditation, tai chi, yoga, and general spiritual/religious activities across various racial/ethnic groups in the United States and internationally. Informing our conceptual model (Figure 1), we identified potential mechanisms across three domains: psychosocial, behavioral, and neurobiological. A total of five studies examined neurobiological mediators (i.e., gene regulation expression, inflammatory markers, immune response, stress markers, and brain efficiency) while twenty‐five studies examined secondary outcomes for consideration as potential mechanisms. These included psychosocial (i.e., quality of life, mindfulness, and perceived stress), behavioral (i.e., anxiety, depression, mood, sleep, functional health, ADLs, and behavioral symptoms), and neurobiological (i.e., glucose levels, stress, synaptic plasticity, mental shifting, cerebral blood flow, and telomerase activity) factors.

**Conclusion:**

Primary mechanisms appeared to focus primarily on neurobiological factors while secondary mechanisms focused on mental health. No identified studies focused exclusively on Black or rural populations who experience greater cognitive disparities. Additionally, no studies used statistical mediation analysis to examine mechanisms that explain the effects of spiritual practice on cognitive health. Rigorous methodological studies are needed to test the underlying mechanisms involved, which might be targets for behavioral interventions.